# Base composition, selection, and phylogenetic significance of indels in the recombination activating gene-1 in vertebrates

**DOI:** 10.1186/1742-9994-6-32

**Published:** 2009-12-16

**Authors:** Ylenia Chiari, Arie van der Meijden, Ole Madsen, Miguel Vences, Axel Meyer

**Affiliations:** 1 Lehrstuhl für Zoologie und Evolutionsbiologie, Department of Biology, University of Konstanz, 78457 Konstanz, Germany; 2 Institut des Sciences de l'Evolution: ISE-M, Université Montpellier 2, 2, Place Eugène Bataillon, CC064, 34095 Montpellier, cedex 5, France; 3 CIBIO, Centro de Investigação em Biodiversidade e Recursos Genéticos, Campus Agrário de Vairão, 4485-661 Vairão, Portugal; 4 Animal Breeding and Genomics Centre, Wageningen University, P. O. Box 338, 6700AH Wageningen, the Netherlands; 5 Division of Evolutionary Biology, Zoological Institute, Technical University of Braunschweig, Spielmannstr. 8, 38106 Braunschweig, Germany

## Abstract

**Background:**

The Recombination Activating Proteins, RAG1 and RAG2, play a crucial role in the immune response in vertebrates. Among the nuclear markers currently used for phylogenetic purposes, *Rag1 *has especially enjoyed enormous popularity, since it successfully contributed to elucidating the relationships among and within a large variety of vertebrate lineages. We here report on a comparative investigation of the genetic variation, base composition, presence of indels, and selection in *Rag1 *in different vertebrate lineages (Actinopterygii, Amphibia, Aves, Chondrichthyes, Crocodylia, Lepidosauria, Mammalia, and Testudines) through the analysis of 582 sequences obtained from Genbank. We also analyze possible differences between distinct parts of the gene with different type of protein functions.

**Results:**

In the vertebrate lineages studied, *Rag1 *is over 3 kb long. We observed a high level of heterogeneity in base composition at the 3^rd ^codon position in some of the studied vertebrate lineages and in some specific taxa. This result is also paralleled by taxonomic differences in the GC content at the same codon position. Moreover, positive selection occurs at some sites in Aves, Lepidosauria and Testudines. Indels, which are often used as phylogenetic characters, are more informative across vertebrates in the 5' than in the 3'-end of the gene. When the entire gene is considered, the use of indels as phylogenetic character only recovers one major vertebrate clade, the Actinopterygii. However, in numerous cases insertions or deletions are specific to a monophyletic group.

**Conclusions:**

*Rag1 *is a phylogenetic marker of undoubted quality. Our study points to the need of carrying out a preliminary investigation on the base composition and the possible existence of sites under selection of this gene within the groups studied to avoid misleading resolution. The gene shows highly heterogeneous base composition, which affects some taxa in particular and contains sites under positive selection in some vertebrate lineages in the 5'-end. The first part of the gene (5'-end) is more variable than the second (3'-end), and less affected by a heterogeneous base composition. However, in some vertebrate lineages the 5'-end of the gene is not yet widely used for phylogenetic studies.

## Background

The majority of recent phylogenetic studies of vertebrates have relied on genetic data of both mitochondrial and nuclear origins (reviewed in [1]). Often, nuclear genes are considered to be superior to mitochondrial ones, especially to resolve deep divergences (e.g., [2]). Furthermore, the use of a single gene, especially if mitochondrial, for phylogenetic reconstructions may not reflect the "true tree" due to a number of reasons, including past hybridization, gene duplication, and/or incomplete lineage sorting. Only a few studies have sought to understand why some nuclear genes are better suited for phylogenetic reconstruction than others ([2,3], but see also [4,5]). Some of the factors that negatively influence the utility of a gene to recover the correct phylogeny include: a heterogeneous base composition [6,7], codon position saturation (reviewed in [1]), and transition/transversion rate bias.

In recent years, DNA sequences of *Rag1 *have been used for phylogenetic inference at various taxonomic levels (e.g., [8-11]). Several studies have focused on specific vertebrate groups (e.g., birds, turtles, sharks and amphibians [8,12-14], but see also [10]) and have highlighted the characteristics of this gene in relation to its phylogenetic utility. Some of these potentially useful characteristics of *Rag1 *include its existence as a single copy gene (except in polyploidy taxa such as *Xenopus*, [15]), uninterrupted exon (except in ray-finned fish where it has one or two introns [16]), the conserved nature of certain regions of the gene, especially its second half (3' end), which facilitates the design of degenerate "universal" primers for PCR, the presence of numerous sequences from a variety of taxa in public databases, and an overall lack of saturation [10].

**Figure 1:**
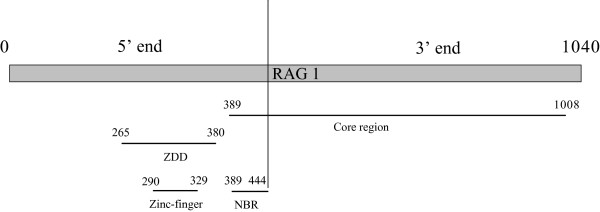
**Organization of RAG1 in *Mus musculus***. Organization of RAG1 (based on *Mus musculus*, P15919 of http://www.uniprot.org/[26]). The vertical line indicates the division in 5' and 3'-ends used in this paper. The analyzed zinc finger and DNA binding domains are indicated. Numbers indicate the amino acid positions in *Mus musculus *RAG1 sequence. "ZDD" indicates the zinc binding domain (which contains the zinc finger domain analyzed in this paper). "NBR" indicates the nonamer binding domain (which corresponds to the DNA binding domain analyzed in this paper). "Core region" indicates the region of the protein needed for cleavage activity (see [17] for additional information).

The protein products of the two lymphocyte-specific recombination activating genes, *Rag1 *and *Rag2*, play an essential role in the host's active immune response to the different pathogens (see [17] and references therein for specific different activity of each protein in the immunological response), starting the process that generates specific receptors on B and T lymphocytes. The immune system is able to target and destroy many different foreign invaders as a result of the vast number of these specific receptors. The specificity of these receptors is made possible by a process known as V(D)J joining. This mechanism occurs in vertebrates and relies on the shuffling and recombination of different pre-existing gene fragments (V (variable), J (joining) and in some case D (diversity)) [17]. The first step of this set of reactions is the recognition and cleavage of a well conserved Recombination Signal Sequence (RSS), consisting of seven or nine nucleotide sequences separated from each other by a spacer of 12 or 23 bp [18]. The *Rag1 *coding sequence contains a conserved protein structural domain that binds the RSS [19]. The active site for the RSS binding and DNA cleavage is contained in part of the so-called the "core RAG1 domain", which also contains the nonamer-binding region (NBR, Figure 1) and three active residues. The recombination process requires that the RAG1 and RAG2 proteins act together as a heterodimer to recognize the RSS (reviewed in [20]) and introduce a break between the RSS and the adjacent V(D)J coding segments [21]. Almost the entire length of RAG1 is involved in codifying for different protein functions (e.g., sites involving in binding RAG2, sites involving in binding the RSS, Figure 1 and [17,22] for additional information on RAG1 structure).

In this study, we focus our attention on the comparative analysis of the variation in the coding region of *Rag1 *within and among major vertebrate clades (Actinopterygii, Amphibia, Aves, Chondrichthyes, Crocodylia, Lepidosauria, Mammalia, Testudines). A few studies on much smaller datasets highlighted possible characteristics of this marker that could affect phylogenetic reconstruction, such as base compositional bias (e.g., [10,23]). The large dataset used in the present work allows us to further study the extent of the base compositional bias across vertebrates as well as other factors that could influence phylogenetic reconstructions. Such a large dataset across vertebrates also allows us to look at the occurrence of indels across vertebrates and to evaluate their utility as phylogenetic characters. Additionally, we also comparatively analyze different parts of the gene, with potential different levels of constraints. We aligned a total of 582 vertebrate sequences yielding an alignment that spans over 3.5 Kb to specifically look at the variability, base composition, selection and utility of indels as phylogenetic characters of *Rag1*.

## Methods

### Datasets

A set of *Rag1 *sequences for each vertebrate clade was downloaded from GenBank in 2008. Most of the downloaded sequences had been used in the framework of published phylogenetic studies. Due to computational limitations, different datasets were used in a few cases. For genetic distances and base compositional analyses, a dataset of 582 vertebrate sequences was used. For the analysis of selection a dataset including a maximum of 60 sequences for vertebrate group was used, while for the analysis of the utility of indels as phylogenetic characters two distinct datasets, one of 1569 and one of 572 sequences, respectively have been used. Each dataset has been built according to the following strategies. Initial clade-wise amino acid alignments of all the downloaded sequences were made manually using MEGA 4.0 [24] using the amino acid sequences. These sub-alignments (312 sequences of Actinopterygii, 181 of Amphibia, 539 of Aves, 30 of Chondrichthyes, 19 of Crocodylia, 150 of Lepidosauria, 313 of Mammalia, and 25 of Testudines), were used only for a more detailed within-clade examination of indels. Of these 1569 downloaded sequences, a subset dataset including 582 vertebrate sequences of *Rag1 *[see Additional file 1] was constructed based on the following characteristics: a) we included one sequence per species. Non-overlapping sequences from the same species were combined to obtain one single sequence. For overlapping sequences of the same species, the longest one was kept, whereas for sequences of the same species of the same length randomly one of the sequences was chosen. b) Sequences shorter than 1000 bp, on the base of each separate within-clade alignment, were removed as well as sequences above this size but not overlapping for a large part (at least 1000 bp) of the gene; in our dataset all the sequences within each vertebrate group span a common fragment of at least 1000 bp. Since numerous studies based on *Rag1 *often used only fragments of the gene, not all the downloaded sequences spanned overlapping regions of the gene and fragments of different lengths were thus included in our dataset (Table 1). c) The remaining sequences were collapsed, to see if identical sequences where present in the dataset, into one single unique nucleotide sequence using the software DNAcollapser v1.0 [25]. d) When possible, we selected a similar number of sequences for each of the major vertebrates groups (110 Actinopterygii, 113 Amphibia, 119 Aves, 84 Lepidosauria, and 89 Mammalia; see Table 1 for the number of sequences of the other groups). To obtain these, we randomly removed sequences using the random number generator as available in Microsoft Excel (version 2003), but making sure that a phylogenetic spread was maintained. We then manually refined the alignment of each clade in MEGA using the amino acid sequences, taking amino acid properties into consideration for optimal alignment. Finally, the complete *Rag1 *sequences [see Additional file 1] were used to check and manually correct the alignment of all vertebrates (alignments available from the authors on request, vertebrate alignment available as additional file, see below indel analysis). Indels were added as indicated in the paragraph on the indel analysis (see below).

**Table 1:** Number of *Rag1 *sequences present in our alignment

	N	Bp	Complete gene	2000 bp <N <total length	1000 bp <N<2000 bp
Actinopterygii	110	3246	2	0	108
Amphibia	113	3252	2	5	106
Aves	119	3141	1	118	0
Chondrichthyes	30	3336	1	0	29
Crocodylia	13	2947	0	12	1
Lepidosauria	84	2928	0	80	4
Mammalia	89	3165	6	31	52
Testudines	24	2793	0	24	0

Number of sequences (N) present in our 582 sequences alignment, divided into three length classes (based on clade-wise alignment and not on the total vertebrate alignment). "Complete" indicates sequences included in the alignment for which the coding region of *Rag1 *has been entirely sequenced (bold numbers, [see Additional file 1]). "2000 bp < N<total length" indicates the number of sequences of more than 2000 base pairs, but not full length. "1000 bp < N<2000 bp" indicates the number of sequences between 1000 and 2000 base pairs long.

The amino- and carboxy-terminals of the gene were also studied separately. To define the sites and regions of the gene with different functional constraint, we used the online site uniprot.org [26]. We divided the gene into two parts of almost equal length (Figure 1) to allow comparable analysis in terms of bp included in the two gene regions. A division of the gene into "core" and "non-core" regions (Figure 1) would have produced a 3'-end being nearly twice as big as the 5'-end (1152 bp versus 1968 bp). Thus, the sequences included in the 582 sequences dataset were split into a 5'-end part (amino acid 1-444 in *Mus musculus*) including a zinc finger, a DNA binding domain and NBR (RSS binding region) regions and a 3'-end part (amino acid 445-1040 in *Mus musculus*), which contains the major part of the core region (Figure 1). This partitioning corresponds also to current practice in phylogenetic studies, where for some vertebrate groups (Actinopterygii, Amphibia, Chondrichthyes, and Mammalia) the commonly used fragment only spans the second half of the gene (generally after amino acid 500). Thus, the division into two separate domains in our study reflects the need of having two similarly long fragments to compare, of which one covers as much as possible the "core-region" of the gene and the other the "non-core" region, and also represents the "most common" gene fragment used for phylogenetic studies in some vertebrate groups. Not every sequence of the 582 dataset spanned both parts of *Rag1*, thus Table 2 indicates the actual number of sequences used for the comparative analysis on the two parts of the gene and their beginning and ending points. Actinopterygii and Chondrichthyes were included in the gene comparison analysis only for the 3'-end, as only two and one of the available sequences, respectively, span the 5'-end of the gene. We furthermore separately analyzed two functional regions of the gene (a zinc binding and DNA binding domains) contained in the 5'-end of *Rag1*, to see if specific functional regions strongly depart from the general characteristics of the gene.

**Table 2:** Number of sequences present in our alignment for each part (5' and 3'-ends) of *Rag1*

		N	Bp	Complete fragment	1000 bp <N <complete fragment
Actinopterygii	3'	110	1453-3246	2	108
Amphibia	5'	7	1-1461	2	5
	3'	113	1462-3252	2	111
Aves	5'	119	1-1353	1	118
	3'	119	1354-3141	1	118
Chondrichthyes	3'	30	1360-3338	1	29
Crocodylia	5'	13	1-1257	2	11
	3'	13	1258-2947	1	12
Lepidosauria	5'	80	1-1263	68	12
	3'	84	1264-2928	24	60
Mammalia	5'	37	1-1377	6	31
	3'	89	1378-3165	6	83
Testudines	5'	24	1-1212	24	0
	3'	24	1213-2793	23	1

Number of sequences (N) included in our dataset for each part (5' and 3'-ends) of the analyzed fragment of *Rag1*. Different fragment lengths are indicated (based on the same alignment as in Table 1). "Bp" indicates the total number of base pairs or the base pairs range for the analyzed fragment after the within group alignment. "Complete fragment" indicates sequences included in the alignment for which the entire part of the gene as indicated under "Bp" was available. "1000 bp < N<complete fragment" indicates the number of sequences of more than 1000 base pairs, but not covering the full length of the fragment. Note that for Actinopterygii and Chondrichthyes only the 3'-end of the gene is indicated. 3' and 5' indicate the 3' and 5'-ends of the gene, respectively. See Methods for any additional information on the gene divisions and data available for each gene division.

### Genetic distances, and base composition analyses

We computed within each vertebrate lineage the Tamura-Nei distances ([27], to account for different base frequencies) for the entire gene, for each part of it (5' and 3'-ends) and for each codon position, using the option of pairwise deletions for missing sites in MEGA 4.0. We used the same software to calculate the amino acid distances (as uncorrected p-distance), the number of variable sites for the nucleotide and amino acid sequences for each vertebrate lineage for the analyzed fragment as well as for the two parts of it.

Base composition for each codon position was calculated in MEGA 4.0. We calculated base composition skew for AT and GC according to the formulas: AT skew = [(A-T)/(A+T)] and GC skew = [(G-C)/(G+C)]. The same software was also used to study the departure from base compositional stationarity for each vertebrate lineage using the Disparity Index test (I_D_-test). Kumar & Gadagkar [28] highlighted how this test is more powerful than the Chi-square in detecting departure from a homogeneous base composition. The disparity index (I_D_) corresponds to the observed compositional difference between two sequences compared to the compositional difference that would be expected under homogeneity. I_D _equals 0 when the homogeneity assumption is satisfied. The significance of a given I_D _value is then assessed using a Monte Carlo approach.

### Statistical analyses

Comparisons on the within group nucleotide and amino acid distances, and on the within group variable nucleotide and amino acid sites between different parts of the gene (5' and 3'-ends and of the two functional regions) were made using a Welch's t-test (test for unequal variance, unequal sample size and independent samples; test run in the R environment, [29]). To avoid any bias associated with the presence of different numbers of sequences in one part of the gene versus the other, we used for this analysis only sequences that spanned both sides of the two gene parts for at least 1000 bp on each side. Discriminant analysis of base composition was run in R on the dataset containing 582 sequences, using each vertebrate lineage as discriminant factor.

### Analysis of selection

Due to computational limitations, we used a subset of the original 582 sequences dataset for the analyses of selection. This subset contained a maximum of 60 sequences for each vertebrate lineage. To build this dataset we first randomly removed different species belonging to the same genus included in the dataset, and continued by randomly removing sequences if necessary to reach the maximum number of 60 sequences. Tests for positive selection were conducted using PAML 4.1 ([30], codeml option) to estimate the synonymous (d_*S*_: silent changes) and nonsynonymous (d_*N*_: amino acid replacement changes) substitution rates and detect instances of positive selection. The d_*N*_/d_*S *_ratio is a measure of the selection acting on the protein. Positive selection is detected when the ratio is >1, indicating a functional benefit to diversify the amino acid sequence. A neutral model (M1a) was compared to a selection model (M2a) and a likelihood ratio test (LRT) was used to determine if positive selection could be rejected. The LRT is performed by taking twice the log-likelihood difference between the models (M2a and M1a) and comparing this to the distribution with degrees of freedom equal to two, as indicated in [31]. Because this method relies on a gene tree, for each vertebrate lineage a NJ tree was created using MEGA 4.0 (trees not shown).

### Analysis of indels

In numerous cases, indels had to be added to correctly align the amino acid sequences. A "correct alignment" was defined as the most parsimonious one, in which indels are added to maintain amino acid blocks in the indel-flanking regions. Due to difficulties in aligning the sequences of all vertebrates, for the analysis of indels on the 582 sequences dataset, the first 315 bp (105 amino acids) of the complete vertebrate alignment were removed from the alignment [see Additional file 2]. We also used the full dataset of 1569 sequences and the entire length available for the different vertebrate groups, to study the exclusivity of these indels within each vertebrate group. Furthermore, to study how phylogenetically informative these indels are within and among the different vertebrate lineages, we coded them to infer a Maximum Parsimony (MP) tree, using PAUP* [32]. The analysis was based on a presence-absence matrix using the amino acid alignment of the 582 sequence dataset without the first 105 amino acids (see above) and codifying indels as 1 and any amino acid as 0. Within some groups (eg., Actinopterygii, Amphibia and Chondrichthyes) only a few sequences span almost the entire length of the gene (Table 1). To avoid misleading results due to the longer fragment represented by these sequences, we removed them from the original dataset (one sequence of Chondrichthyes, two of Actinopterygii and seven of Amphibia). Thus, the total number of sequences used was 572.

## Results

### Comparison across vertebrates

Table 1 shows the distribution in terms of length of the sequences used for each vertebrate lineage. Table 2 shows the same distribution for each part of the gene studied separately. Nucleotide substitution varies across sites, with the 3^rd ^codon position being more variable than the other two [see Additional file 3]. The pattern of variation in terms of genetic distances is, as expected, 3^rd^>1^st^>2^nd^.

Table 3 shows, for each group, the number of variable sites in *Rag1*, indicating that Amphibia, and Lepidosauria have the highest percentage of variable nucleotide sites across the entire gene and at each codon position. Amphibia and Lepidosauria also have the highest percentage of variable amino acid sites. The higher variability observed in Aves, Lepidosauria, and Mammalia at the third codon position, compared to taxa of older origin like Actinopterygii, is probably caused by differences in number of sequences spanning also the 5'-end of the gene (37 or more sequences compared to zero, Table 2) included in the analysis (see below for variability of 5' and 3'-ends of the gene). In fact, when considering the 3'-end of the gene, nucleotide and amino acid variability in Actinopterygii is higher or comparable to the above mentioned groups (Table 3).

**Table 3:** Variable nucleotide and amino acid sites in *Rag1*

		N	Bp	Variable nucleotides	Variable nucleotide 1^st ^pos	Variable nucleotide 2^nd ^pos	Variable nucleotide 3^rd ^pos	N aa	Variable aa
Actinopterygii	F	110	3246	46	38	27	74	1082	43
	3'	110	1453-3246	56	47	31	90	598	50

Amphibia	F	113	3252	60	50	38	92	1084	57
	5'	7	1-1461	62	51	45	89	487	60
	3'	113	1462-3252	58	50	32	94	597	55

Aves	F	119	3141	48	39	23	86	1047	39
	5'	119	1-1353	59	48	41	87	451	58
	3'	119	1354-3141	39	23	10	85	596	25

Chondrichthyes	F	30	3336	19	13	9	36	1112	16
	3'	30	1360-3336	32	22	15	54	659	26

Crocodylia	F	13	2947	5	5	1	9	983	5
	5'	13	1-1257	6	5	2	10	419	8
	3'	13	1258-2947	4	4	1	8	564	4

Lepidosauria	F	84	2928	63	53	41	97	976	58
	5'	80	1-1263	75	67	61	98	421	74
	3'	84	1264-2928	54	42	25	96	555	45

Mammalia	F	89	3165	52	38	27	92	1055	42
	5'	37	1-1377	61	50	42	92	459	61
	3'	89	1378-3165	45	28	15	93	596	28

Testudines	F	24	2793	26	16	10	53	931	20
	5'	24	1-1212	31	23	16	52	404	30
	3'	24	1212-2793	23	10	5	53	527	11

The variability in nucleotides and amino acids is presented in percentages (rounded to the higher integer) for the 582 sequences dataset. "N" indicates the number of sequences included. "Bp" indicates the total number of base pairs or the base pairs range for the analyzed fragment after the within group alignment. "N aa" indicates the number of amino acids in the analyzed fragment after the within group alignment. Note that for Actinopterygii and Chondrichthyes only the 3'-end of the gene is indicated. "F", "5"' and "3"' indicate the full gene and the 5' and 3'-ends of the gene, respectively. See Methods for any additional information on the gene divisions and data available for each gene division.

**Figure 2:**
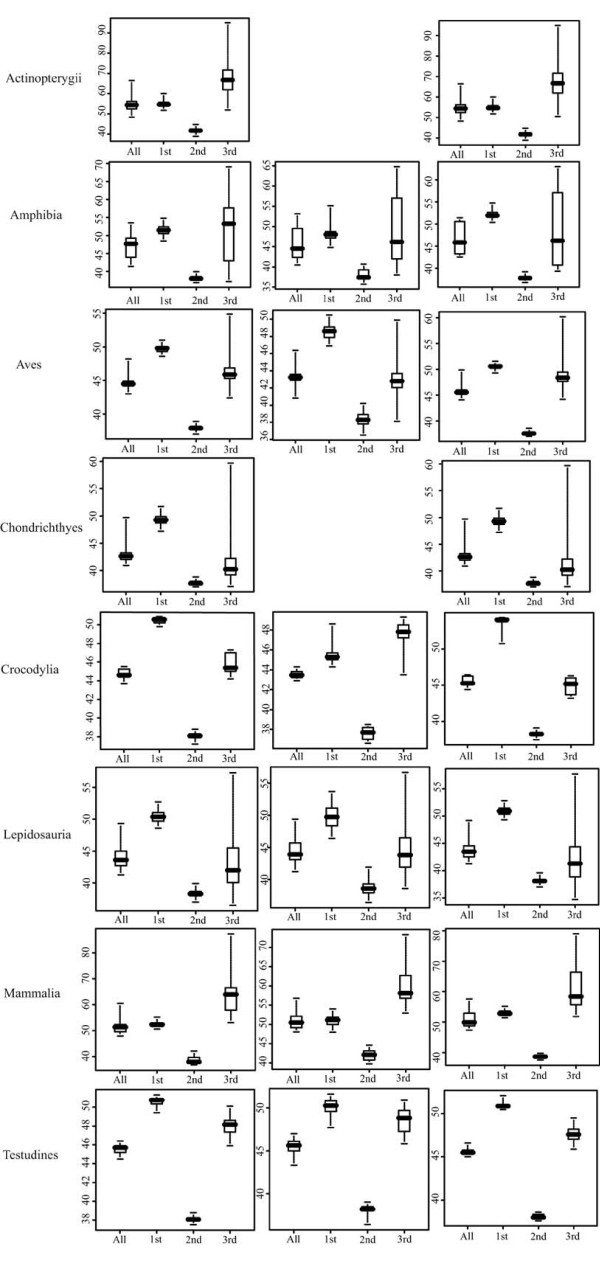
**Boxplot of GC content of *Rag1***. Boxplot of the GC content (in %) considering all positions, 1^st^, 2^nd ^and 3^rd ^position, respectively in the entire studied fragment and in the 5' and 3'-ends for each vertebrate lineage. Whiskers represent most extreme values. Rectangles are made of 1^st ^and 3^rd ^quartiles. Horizontal black bar represents median value.

**Figure 3:**
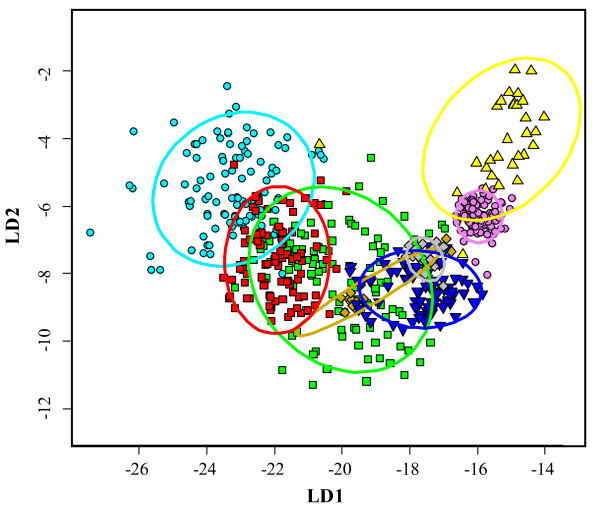
**Discriminant analysis of total base composition of *Rag1***. Results of the linear discriminant analysis along the first and second discriminant axes (LD1 and LD2, respectively). LD1 and LD2 account for 80% and 18% discriminant power, respectively. 95% probability ellipses are indicated for each group. Each color corresponds to a different lineage. Color code is similar to the one in Figure 4. Light blue circle = Actinopterygii, green square = Amphibia, violet circle = Aves, yellow up triangle = Chondrichthyes, gold diamond = Crocodylia, blue down trianle = Lepidosauria, red square = Mammalia, grey diamond = Testudines.

Table 4 shows the results of the analysis of base composition for each vertebrate lineage. In *Rag1*, the hypothesis of homogeneous base frequencies is rejected at the 3^rd ^codon position (p < 0.05) for more than half of the pairwise comparisons within several vertebrate lineages (Actinopterygii, Amphibia, Lepidosauria and Mammalia, Table 4). Moreover, lineages that do not show an overall high departure from homogeneous base composition (Aves, Chondrichthyes and Testudines, but not Crocodylia), still have species showing an especially strong heterogeneous base composition compared to the rest of the within lineage dataset (e.g., in Aves *Schiffornis turdinus, Sitta pygmaea *and *Sitta carolinensis*, data not shown). An analysis of base compositional skew reveals a general high level of A versus T, and G versus C (adenine versus thymine and guanine versus cytosine) at the 1^st ^codon position, a general skew versus A and C at the 2^nd ^codon position and no general trend at the 3^rd ^codon position [see Additional file 4]. Base composition for the gene does not reflect a general trend (all values are rounded up, T = 24%, C = 22%, A = 29%, G = 25%). CG frequency is highly variable at the 3^rd ^codon position in Amphibia (Figure 2), even if the mean values for the gene are very similar across the vertebrate lineages [see Additional file 4]. The lineages for which GC frequency does not show high variation still have some sequences with high departure from the common variation range (indicated by the whiskers, Figure 2). This is especially true for Chondrichthyes and Aves. Furthermore, in a discriminant analysis on the total base composition Actinopterygii and Chondrichthyes distinguish well from the remaining vertebrate groups along the first and second discriminant axes, respectively (Figure 3).

**Table 4:** Proportion of *Rag1 *sequences for which the homogeneity assumption is rejected.

		N	Bp	P < 0.05			
				
				Total	1^st^	2^nd^	3^rd^
Actinopterygii	F	110	3246	63	18	5	61
	3'	110	1453-3246	63	25	2	62

Amphibia	F	113	3252	65	10	3	66
	5'	7	1-1461	52	29	0	67
	3'	113	1462-3252	65	11	3	65
	ZF	7	991-1110	43	5	5	48
	DB	7	1294-1461	48	10	0	43

Aves	F	119	3141	40	9	8	40
	5'	119	1-1353	27	8	6	27
	3'	119	1354-3141	31	10	7	33
	ZF	119	883-1002	3	0	0	2
	DB	119	1186-1353	15	0	0	15

Chondrichthyes	F	30	3336	29	11	4	33
	3'	30	1360-3336	31	12	4	34

Crocodylia	F	13	2949	27	3	27	32
	5'	13	1-1257	0	4	0	0
	3'	13	1258-2949	27	0	24	23
	ZF	13	787-906	0	0	0	0
	DB	13	1090-1257	5	0	0	8

Lepidosauria	F	84	2928	53	10	5	51
	5'	80	1-1263	34	11	3	30
	3'	84	1264-2928	46	3	3	50
	ZF	80	793-912	12	3	1	12
	DB	80	1096-1263	14	3	0	16

Mammalia	F	89	3165	54	9	7	54
	5'	37	1-1377	46	15	16	40
	3'	89	1378-3165	54	6	6	54
	ZF	37	913-1032	17	10	0	15
	DB	37	1210-1377	26	4	0	23

Testudines	F	24	2793	21	8	7	12
	5'	24	1-1212	12	11	6	28
	3'	24	1213-2793	13	11	3	2
	ZF	24	742-861	1	0	0	1
	DB	24	1045-1212	7	10	0	1

Proportion of sequences (in % rounded up) of *Rag1 *for each group for which the assumption of homogeneous base composition is rejected using the disparity index test (p < 0.05; Monte Carlo test with 500 replicates). "N" indicates the number of sequences used. Alignment based on the 582 sequences dataset. "Bp" indicates the total number of base pairs or the base pairs range for the analyzed fragment after the within group alignment. Note that for Actinopterygii and Chondrichthyes only the 3'-end of the gene is indicated. "F", "5"' and "3"' indicate the full gene and the 5' and 3'-ends of the gene, respectively. "ZF" and "DB" indicate the zinc finger and DNA binding domains, respectively. See Methods for any additional information on the gene divisions and data available for each gene division.

Table 5 shows the results of the selection test run on the different vertebrate groups. The d_N_/d_S _ratio was always lower than one, supporting the neutral selection scenario along the entire gene. However, the results of the chi-square test do not reject positive selection acting at some sites in Aves and Lepidosauria (Table 5). Moreover, we found positively selected sites in Aves, Lepidosauria and Testudines in the 5'-end of the gene. According to the human sequence of RAG1 on uniprot.org [26], these sites occur in a region with functional activity (the interaction with importin alpha-1).

**Table 5:** Selection test

		Log-likelihood	d_*N*_/d_*S*_	P-value Chi-square	PSS
Actinopterygii	Log-likelihood M1a	-21118.741643	0.1331	1	-
	Log-likelihood M2a	-21118.741643	0.1331		
Amphibia	Log-likelihood M1a	-22490.686927	0.1049	1	-
	Log-likelihood M2a	-22490.686928	0.1049		33 T
Aves	Log-likelihood M1a	-16728.712033	0.1695	10^-7^*	76 W
	Log-likelihood M2a	-16714.234740	0.1807		
Chondrichthyes	Log-likelihood M1a	-6993.815583	0.1783	1	-
	Log-likelihood M2a	-6993.815583	0.1783		
Crocodylia	Log-likelihood M1a	-3538.892722	0.2144	1	-
	Log-likelihood M2a	-3538.892722	0.2144		
Lepidosauria	Log-likelihood M1a	-38632.722264	0.2632	0.032*	159 H
	Log-likelihood M2a	-38629.286234	0.2665		
Mammalia	Log-likelihood M1a	-10175.360951	0.0713	1	-
	Log-likelihood M2a	-10175.360951	0.0713		
Testudines	Log-likelihood M1a	-10208.575616	0.1653	0.1145	23 V
	Log-likelihood M2a	-10206.408516	0.1730		

Test for positive selection using the M1a (neutral) and M2a (positive) models, and a maximum of 60 *Rag1 *sequences for each vertebrate lineage (see Methods for further explanations). "d_*N*_/d_*S*_" indicates the substitution rate ratio averaged across all sites and lineages. "PSS" indicates the positively selected sites (calculated as in [47]). We indicate the position of the amino acid under positive selection (based on the within group alignment used for this analysis) and the corresponding amino acid. * indicates the statistically significant p-values (<0.05) in the Chi-square test.

### Analysis of indels across vertebrates

**Figure 4:**
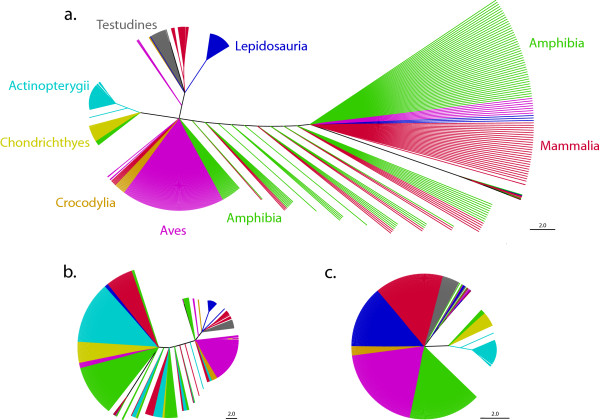
**Maximum Parsimony tree (MP) of indels in *Rag1 *sequences**. Maximum Parsimony strict consensus tree (MP) of the indels, using a 572 sequences dataset of 1076 amino acids. Different colors correspond to different vertebrate lineages, as indicated in the text of this figure and in Figure 3. The scale bar next to each tree refers to the branch length and indicates the numbers of amino acid substitutions per site. **a) **MP based on the entire analyzed fragment. **b) **MP based on the 5'-end of the gene. **c) **MP based on the 3'-end of the gene.

Figure 4 shows a strict consensus MP cladogram based on the indels (homoplasy index: 0.607143; tree length 280). Although indels confer some information to identify higher taxa, only Actinopterygii could be resolved as a monophyletic group. All lepidosaurs are united into one clade, with the exception of single sequences of the worm lizards *Blanus*, *Bipes*, the lizard *Dibamus *and the snake *Loxocemus*. These four sequences are not recovered in a clade with the remaining Lepidosauria probably due to their short sequence length, lacking up to 478 amino acid positions in the 5'-end of the gene relative to the rest of the alignment. The lizard *Lanthanotus *was placed outside the main Lepidosauria clade based on a single indel at position 370 of the amino acid alignment.

**Figure 5:**
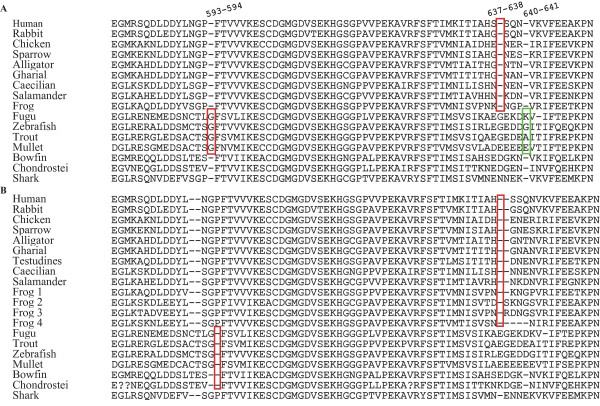
**Indels Alignment**. Alignment of a fragment of *Rag1 *based on **A) **Venkatesh and colleagues [43] and **B) **the alignment used in this work. Numbers on the alignment are according to [43]. Red boxes indicate synapomorphic indels according to [43]. The green box indicates the indel identified as synapomorphic by [43] occurring in a highly variable region and not recovered as synapomorphic according to our alignment. Sequences of **A) **are as in [43]. Sequences in **B) **(see [Additional file 1] for species names) Human: M29474; Rabbit: M77666; Chicken: M58530; Sparrow: AF143738; Alligator: AF143724 AY125022; Gharial: AF143725; Testudines AY687922; Caecilian EF551566; Salamander AY583351; Frog 1: L19324; Frog 2: AY323762; Frog 3: AY323771; Frog 4: AY571659; Fugu: AY700363; Zebrafish: U71093; Trout: U15663; Mullet: AY308783; Bowfin: AY430199; Chondrostei: AY430198; Shark: U62645. Names and species used in the Figure have been choosing as in [43]. We added to the alignment in **B) **only a few sequences which help to show the higher variability of certain indels regions compared to the **A) **alignment.

Within each major vertebrate group, insertions and deletions often seem to be characteristic of a subset of genera, indicating possible monophyletic subgroups. In Actinopterygii, an amino acid deletion (amino acid position 612 in the alignment, [see Additional file 2]) occurs in two of the four families (Tetraodontidae and Diodontidae) of the suborder Tetraodontoidei (as already reported in [33]). In Amphibia, representatives of a derived clade in the superfamily Hyloidea [34,35] have an amino acid insertion (in position 606 of the alignment, [see Additional file 2]) as already reported by [36], which is lacking in basal hyloids such as *Caudiverbera, Lechriodus*, *Limnodynastes*, and *Myobatrachus*, and in all the other amphibian species included in our dataset. This insertion occurs in a variable region (see Discussion relative to Figure 5 of this manuscript) and also occurs in Chondrichthyes, probably convergently. All Aves, except some genera of chicken and duck-like birds and paleognaths (*Aburria*, *Anas*, *Anhima*, *Chamaepetes*, *Chauna*, *Crax*, *Gallus*, *Megapodius*, *Mitu*, *Nothocrax*, *Oreophasis*, *Orthalis*, *Pauxi*, *Penelope*, *Penelopina*, *Pipile*, *Struthio*, and *Tinamus*; see [8,37] for the phylogeny) have a deletion of five amino acids that it is absent in other vertebrate groups (amino acid positions 7-11 in the alignment, [see Additional file 2], these amino acids occur in a variable region across vertebrates). In the superfamily Passeroidea (Aves) an insertion of four amino acids (amino acid positions 201-204 in the alignment, [see Additional file 2]) is characteristic of the monophyletic group called "core Passeroidea" and includes the genera *Cardinalis*, *Emberiza*, *Estrilda*, *Fringilla*, *Icterus*, *Motacilla*, *Parula*, *Passer*, *Ploceus*, *Prunella*, and *Thraupis *(see [38] for phylogeny). In the order Passeriformes (Aves) a deletion of one amino acid (amino acid position 336 in the alignment, [see Additional file 2]) seems to have evolved multiple times as it occurs in some genera not belonging to a monophyletic group (*Ptilonorhynchus*, *Dryoscopus *and *Ailuroedus*, for large avian phylogeny see [39]). In Testudines, the family Emydidae shows a deletion of three amino acids that seems to be characteristic of the clade including the genera *Graptemys *and *Trachemys*, but not shared by the other Emydidae included in our dataset (*Actinemys*). Finally, synapomorphic insertions or deletions characterize a specific genus (e.g., an insertion of 20 amino acids in *Corvus *(Aves)), a subfamily (e.g., in Amphibia the only two genera of the subfamily Rhacophorinae included in our dataset, *Philautus *and *Polypedates*, have a deletion of four amino acids), or a family (e.g., an amino acid insertion characteristic of all the genera belonging to the family Bombycillidae in Aves and a deletion of six amino acid characteristic of the family Chamaeleonidae (Lepidosauria)), or in the parvorder Anguimorpha (Lepidosauria) (a single amino acid insertion).

### Comparison of different regions of *Rag1*

The 5' and 3'-ends of the gene differ in terms of genetic diversity, with the 5' significantly more variable than the 3', across all vertebrates (Table 6) as well as within single vertebrate lineages, with the exception of the number of variable nucleotide sites for Aves, Lepidosauria (but for these two groups the p-value is very close to significance), Mammalia, and Testudines (Table 7).

**Table 6:** Comparative analyses across vertebrates of the 5' and 3'-ends of *Rag1*

	*t*	df	p-value
Tamura-Nei nucleotide distances	4.8294	22	7.969e-05*
Number of variable nucleotide sites	5.6585	22	1.087e-05*
AA p-distances	3.3504	4	0.02856*
Number of variable aa	4.0853	4	0.01503*

Comparative analyses across vertebrates of the two parts of *Rag1*, the 5' and 3'-ends. Number of sequences used for each monophyletic vertebrate lineage: Amphibia = 7; Aves = 119; Crocodylia = 13; Lepidosauria = 80; Mammalia = 37; Testudines = 24. "df" indicates the degree of freedom. * indicates the significant p-value (p < 0.05).

**Table 7:** Comparative analysis for each vertebrate group of the 5' and 3'-ends of *Rag1*

	Variables	*t*	df	p-value
Amphibia	Tamura-Nei nucleotide distances	17.4	3	0.0004137*
	Number of variable sites	3.4447	3	0.04110*
Aves	Tamura-Nei nucleotide distances	5.7446	3	0.01048*
	Number of variable sites	3.1192	3	0.05251
Crocodylia	Tamura-Nei nucleotide distances	-	-	-
	Number of variable sites	5.1962	3	0.01385*
Lepidosauria	Tamura-Nei nucleotide distances	4.8414	3	0.01681*
	Number of variable sites	3.1114	3	0.05283
Mammalia	Tamura-Nei nucleotide distances	6.6541	3	0.006918*
	Number of variable sites	2.9613	3	0.05948
Testudines	Tamura-Nei nucleotide distances	9	3	0.002896
	Number of variable sites	2.5062	3	0.08723

Comparative analysis within each vertebrate lineage of the two parts of *Rag1*, 5' and 3'-ends. For each vertebrate lineage average Tamura-Nei nucleotide distances, and average number of variable nucleotide sites within group have been used for the analysis. Tamura-Nei nucleotide distances cannot be compared for Crocodylia as data are essentially constant between the two parts of the gene. Number of sequences used for each monophyletic vertebrate lineage: Amphibia = 7; Aves = 119; Crocodylia = 13; Lepidosauria = 80; Mammalia = 37; Testudines = 24. "df" indicates the degree of freedom. * indicates the significant p-value (p < 0.05).

The two functional regions included in the 5'-end of the gene do not differ significantly in terms of genetic distances (Table 8). Base composition analysis of the two parts of the gene indicates that GC content in the two parts of the gene in general is similar to what has been observed for the total analyzed gene (Figure 2). Homogeneous base frequencies is rejected at 3^rd ^codon position (p < 0.05) in about half of the pairwise comparisons in the 5' and 3'-ends of Amphibia and 3'-end of Actinopterygii, Lepidosauria and Mammalia (Table 4).

**Table 8:** Comparative analysis for each vertebrate group of two functional regions of *Rag1*

	*t*	df	p-value
Amphibia	0.3780	3	0.7306
Aves	0.3974	3	0.7177
Crocodylia	1	3	0.3910
Lepidosauria	0.4899	3	0.6578
Mammalia	0.6765	3	0.5472
Testudines	2.4495	3	0.09172

Comparative analysis for each vertebrate group of the Tamura-Nei nucleotide distances between the two functional regions (zinc finger and DNA binding) contained in the 5'-end of the gene. Number of sequences used for each monophyletic vertebrate lineage: Amphibia = 7; Aves = 119; Crocodylia = 13; Lepidosauria = 80; Mammalia = 37; Testudines = 24. "df" indicates the degree of freedom. * indicates the significant p-value (p < 0.05).

Almost all phylogenetically informative indels uniting the Testudines and the Lepidosauria were found in the 5'-end of the gene (Figure 4b), whereas the Actinopterygii were defined as a clade based on indels in the 3'-end of the gene (Figure 4c).

## Discussion

The increasing number of available sequences in GenBank and phylogenetic studies on different groups of organisms has still not solved the main difficulties that researchers have to continuously face to find appropriate phylogenetic markers. This problem is even stronger when the phylogenetic analysis involves distinct taxonomic groups of different divergence times. Numerous articles (e.g., [1,40,41]) have dealt with the factors influencing the reliability of a molecular phylogeny. The lymphocyte-specific recombination activating gene, *Rag1*, has been shown to be a good phylogenetic marker in recovering phylogenetic relationships within and among vertebrate lineages (see Background). However, a few studies have also highlighted some possible flaws of this marker (e.g. in birds [42] and in mammals [23]). The fact that *Rag1 *has become of standard use in vertebrate phylogeny urges the identification of possible problems inherent to this marker.

*Rag1 *shows a strong departure from homogenous base composition (Table 4) and high GC variation at 3^rd ^codon position in some vertebrate lineages and in some taxa within the other vertebrate lineages that do not show an overall high GC variation at this codon position. Moreover, while Hugall and colleagues [10] observed base heterogeneity only at the 3^rd ^codon position in a restricted vertebrate dataset, our results also reveal some departure from homogeneity occurring at the 1^st ^and 2^nd ^position (Table 4) and at the 3^rd ^position in some species. Furthermore, GC content is higher at the 1^st ^codon position than at the 2^nd ^or 3^rd ^position in some lineages (Aves, Chondrichthyes, Crocodylia, Lepidosauria and Testudines, Figure 2). Overall base compositional skew is also a common phenomenon in vertebrates *Rag1*, occurring at 1^st ^and 2^nd ^codon positions [see Additional file 4]. Asymmetries in the base compositions are also reflected by the amino acid use, suggesting a selection for preferred amino acids in the protein. Further studies investigating the 2D and 3D structure of RAG1 in distinct vertebrates could offer insights on the possible selective constraints acting on the base composition. Base composition heterogeneity is thus a relatively common phenomenon in *Rag1 *in vertebrates, not only limited to some vertebrate groups (e.g., [23,42]) and may also influence substitution rate differences [10] and thereby phylogenetic analyses. However, different phylogenetic analyses can be differently affected by compositional bias, as for example on smaller datasets this phenomenon could possibly not be observed. We thus suggest checking for possible base composition asymmetries before to run any phylogenetic analysis using *Rag1*.

Our data also indicate instances of positive selection in some vertebrate lineages (Aves, Lepidosauria) and at one site in Testudines. These sites that are possibly under positive selection occur in a region coding for a unit with functional activity in human RAG1, and further investigation is necessary to understand if this activity is also present in the other major vertebrate lineages. Those lineages in which positive selection has not been observed do not have a large number of sequences spanning the 5'-end of the gene (except for Mammalia), where the positive selected sites occur, indicating that sites under selection may also be present in other lineages, beside the ones observed here. While in the observed case, positive selection at these sites should not influence phylogenetic reconstruction as it does not happen independently (for the same sites and same amino acid changes) on different branches, a more complete dataset across vertebrates would give further insight into the functional activity of these sites within and among vertebrate lineages.

Finally, insertions and deletions are relatively rare genomic events and have hence often been used as synapomorphic characters to resolve evolutionary relationships [43]. Our dataset indicates that insertions and deletions in *Rag1 *may often occur in particular regions of the gene and generally independently among major vertebrate taxa. The phylogenetic reliability of indels may therefore be obscured by homoplasious insertion and deletion events (e.g, as some of the indels in the cellular myelocytomatosis oncogene, c-myc [44]). However, within each vertebrate lineage these derived "length-characters" seem to be phylogenetically informative albeit taxonomically limited (see also Figure 4). Even if our dataset recovered some vertebrate clades, it shows that the presence of indels characteristic of clades is limited within each major vertebrate group, as described for other genes in much smaller datasets (e.g., [44]). Further studies including large datasets, multiple genes and statistical analyses of ancestral state reconstruction, could add further understanding of the frequency with which insertions and deletions occur as homoplasious characters. However, in our large *Rag1 *dataset we did not find clear insertions or deletions that would support the hypothesis of common homoplasy. In fact, indels either occurred at different positions in different groups, or in hypervariable regions of the gene which in any case are difficult to align. Especially in such hypervariable regions, only a large data set across vertebrates would allow distinguishing indels as synapomorphic versus homoplasious, since the presence of certain taxa could change the alignment of the dataset and thus suggest different conclusions. Venkatesh and colleagues [43] identified three single amino acid deletions assumed to be specific for some of the vertebrate lineages analyzed in their alignment of a short fragment of *Rag1*. In comparison to our alignment, however, their synapomorphic characters are not unambiguous and the one in position 637-641 (Figure 5) occurs in a highly variable region. Although phylogenetic results based on the use of single amino acid deletions and insertions as synapomorphies with only limited taxon sampling cannot be generalized (see also discussion in [45]), this does not invalidate the interpretation of major deletions or insertions of longer gene sections as rare genomic events of high phylogenetic significance as observed for example in *Rag1 *in squirrels by Steppan and colleagues [44] (see review and Box 1 in [46], but also [45]).

## Conclusions

*Rag1 *has been demonstrated to be a powerful marker for phylogenetic reconstruction in vertebrates. Its quality is attested by its widespread use in the community of molecular phylogeneticists, and by the quality of the results which are usually in full agreement with those obtained from other, comprehensive data sets. The gene is easily aligned even across divergent taxa of vertebrates, largely appears to lack saturation and does not contain introns in the majority of vertebrates. However, our work and previous studies with a more restricted sampling report on a highly heterogeneous base composition, especially at at the 3^rd ^codon position and for some specific taxa that may affect phylogenetic reconstruction. Moreover, our data also indicate instances of selection in the 5'-end of the gene. Based on our results, the 5'-end of the gene shows higher variability than the 3'-end and it is also slightly less affected by a base composition bias. This suggests that the use of the entire gene length fragment or of the 5'-end may offer better resolution to problematic phylogenetic reconstruction. Especially when controversial and poorly supported relationships are to be studied we suggest verifying if any taxa included in the dataset may show a strong departure from a homogeneous base composition. Finally, our data shows that in *Rag1 *indels do not seem to be generally affected by homoplasy and may be used as informative characters within vertebrate lineages.

## List of Abbreviations

*Rag1: *Recombination Activating Gene-1; *Rag2: *Recombination Activating Gene-2; RAG1: Recombination Activating Gene-1 protein; RAG2: Recombination Activating Gene-2 protein; RSS: Recombination Signal Sequence; NBR: Nonamer-Binding Region; Bp: base pairs; MP: Maximum Parsimony; NJ: Neighbor Joining.

## Declaration of competing interests

The authors declare that they have no competing interests.

## Authors' contributions

YC, OM, MV and AM conceived the study. YC and AvdM carried out the analyses and prepared the figures. YC wrote the manuscript. All authors read and approved this version of the manuscript.

## Supplementary Material

Additional file 1 **Used sequences**. Genbank accession number and species name for each sequence of the 582 sequences dataset. More than one Genbank accession number indicates that multiple sequences for the same species have been merged together (see Methods for additional explanations). Bold numbers indicate full-length sequences. Click here for file

Additional file 2 **Alignment of the 582 vertebrate *Rag1 *sequences**. Alignment of the 582 vertebrate sequences used in this work. The first 105 amino acid have been removed from the alignment (see Methods for additional explanations). Click here for file

Additional file 3 **Average nucleotide and amino acid distances**. **a) **Average Tamura-Nei nucleotide distances, and average amino acid p-distances calculated for the 582 sequence dataset and for each part (5' and 3'-ends) of the entire fragment length of the gene. "N" indicates the number of sequences used. Check Table 1 to see the length range of the sequences included. "Bp" indicates the number of base pairs in the fragment analyzed after the within group alignment. Note that for Actinopterygii and Chondrichthyes only the 3' -end of the gene is provided. "F", "5"' and "3"' indicate the full gene and the 5' and 3' -ends of the gene, respectively.**b) **Average Tamura-Nei nucleotide distances, and amino acid p-distances for the functional zinc-finger and DNA binding domains in the 5' -end of the gene. "ZF" and "DB" indicate the zinc finger and DNA binding domains, respectively (Figure 1). See Methods for any additional information on the gene divisions and data available for each gene division. Click here for file

Additional file 4 **AT and GC skew**. Average GC content (in % round up to higher integer) and base compositional skew for each codon position for each vertebrate group, based on the 582 sequences alignment. For the complete alignment across vertebrates 582 sequences have been used without the first 105 amino acid. See Methods for additional information about how the base compositional skew has been calculated. Click here for file
