# Supplementary material for: Base composition, selection, and phylogenetic significance of indels in the recombination activating gene-1 in vertebrates
Source: Front Zool. 2009 Dec 16;6:32. doi: 10.1186/1742-9994-6-32 (PMC2803162; doi:10.1186/1742-9994-6-32)
Supplement: Additional file 1 — Used sequences. Genbank accession number and species name for each sequence of the 582 sequences dataset. More than one Genbank accession number indicates that multiple sequences for the same species have been merged together (see Methods for additional explanations). Bold numbers indicate full-length sequences. [file 1742-9994-6-32-S1.DOC]

**Additional file 1: Used sequences.** Genbank accession number and species name for each sequence of the 582 sequences dataset. More than one Genbank accession number indicates that multiple sequences for the same species have been merged together (see Methods for additional explanations). Bold numbers indicate full-length sequences.

**Actinopterygii**

*AY208621 Abudefduf sordidus, AY208622 Abudefduf taurus, AY308781 Allocyttus verrucosus, AY700331 Aluterus scriptus, AY308793 Amanses scopas, AY208627 Amblyglyphidodon aureus, AY430199 Amia calva, AY208628 Amphiprion akindynos, AY208629 Amphiprion allardi, AY208630 Amphiprion perideraion, AY208631 Amphiprion ocellaris, AY700346 Anoplocapros inermis, AY700348 Aracana ornata, AY430228 Argentina sialis, AY700349 Arothron nigropunctatus, AY330966 Badis assamensis, AY330967 Badis badis, AY330969 Badis corycaeus, AY330976 Badis sp., AY700309 Balistes polylepis, AY700310 Balistes vetula, AY700320 Balistoides viridescens, AF519702 Betta edithae, AF519705 Betta fusca, AF519710 Betta waseri, AF519712 Betta hipposideros, AF519718 Betta tussyae, AF519725 Betta foerschi, AF519726 Betta simorum, AF519728 Betta spendens, AF519730 Betta patoti, AY700337 Brachaluteres jacksonianus, AY430219 Brachygalaxias bullocki, AY700350 Canthigaster jactator, AY700351 Canthigaster rostrata, AY700352 Canthigaster valentini, AY700366 Canthigaster janthinoptera, AY308775 Chaetodon striatus, AY430220 Chlorophthalmus sp., AY700322 Chilomycterus antenna, AY208634 Chromis retrofasciata, AY208636 Chromis multilineata, AY208637 Chromis atrilobata, AY208638 Chromis nitida, AY208641 Chromis agilis, AY208642 Chromis weberi, AY208645 Chrysiptera annulata, AY208646 Chrysiptera rollandi,* ***U71093*** *Danio rerio,* ***U15663*** *Oncorhynchus mykiss, AY330977 Dario dario, AY330978 Dario hysginon, AY208651 Dascyllus marginatus, AY208653 Dascyllus melanurus, AY208654 Dascyllus reticulatus, AY208655 Dascyllus trimaculatus, AY700327 Diodon liturosus, AY308791 Diodon hystrix, AY208656 Dischistodus melanotus, AY846498 Elacatinus digueti, AY846519 Elacatinus lori, AY846530 Elacatinus evelynae, AY430205 Engraulis japonicus, AY380541 Esox americanus, AY380543 Esox masquinongy, AY380544 Esox niger, AY430226 Etheostoma caeruleum, AY846563 Gobiosoma chiquita, AY438703 Gonostoma bathyphilum, AY208615 Embiotoca lateralis, AY208617 Halichoeres melanurus, AY430200 Hiodon alosoides, AY700344 Lactophrys triqueter, AY700345 Lactophrys quadricornis, AY308764 Lampris guttatus, AF519737 Macropodus opercularis, AY308792 Masturus lanceolatus, AY700313 Melichthys niger, AY430225 Menidia menidia, AY700328 Mola mola, AY700335 Monacanthus hispidus, AY700364 Monotreta leiurus, AY308783 Mugil curema, AY700340 Nelusetta ayraudi, AY208658 Neoglyphidodon nigroris, AY208659 Neoglyphidodon oxyodon, AY208661 Neopomacentrus filamentosus, AY700339 Oxymonacanthus longirostris, AF519740 Parosphromenus deissneri, AY308768 Perca flavescens, AY308774 Peristedion miniatum, AY308765 Polymixia japonica, AY208665 Pomacentrus bankanens, AY208668 Pomacentrus australis, AY208669 Pomacentrus moluccensis, AY208670 Pomacentrus smithi, AY430213 Prosopium williamsoni, AY430222 Regalecus glesne, AY846560 Risor ruber, AY380535 Salvelinus malma, AY430223 Sargocentron punctatissimum, AY308770 Sargocentron vexillarium, AY430198 Scaphirhynchus albus, AY308795 Sphoeroides dorsalis, AY700363 Takifugu rubripes, AY380537 Thaleichthys pacificus, AY380548 Umbra limi, AY380547 Umbra krameri, AY380549 Umbra pygmae, AY308776 Zebrasoma scopas.*

**Amphibia**

*AY323765 Agalychnis callidryas, AY323755 Alytes muletensis, AY583334 Alytes obstetricans, AY650131 Ambystoma gracile, AY650130 Ambystoma opacum, AY583345 Ambystoma ordinarium, AY650127 Amphiuma means, AY571641 Amnirana lepus, AY650142 Andrias davidianus, AY583346 Andrias japonicus, AY691701 Aneides aeneus, AY650118 Aneides lugubris, AY650146 Ascaphus montanus, AY323754 Ascaphus truei, AY650126 Batrachoseps major, AY650124 Bolitoglossa helmrichi, AY571643 Boophis doulioti, AY323756 Bombina orientalis, AY571644 Breviceps fuscus, AY323762 Bufo bufo, AY323763 Bufo regularis, AY583337 Caudiverbera caudiver, AY583347 Chioglossa lusitanica, AY650141 Cryptobranchus alleganiensis, AY571647 Dermatotonus muelleri, AY650148 Dermophis mexicanus, AY691697 Desmognathus brimleyorum, AY650117 Desmognathus quadramaculatus, AY691699 Desmognathus wrighti, AY691695 Dicamptodon copei, AY650132 Dicamptodon tenebrosus, AY583338 Discoglossus galganoi, AY323757 Discoglossus sardus, AY571648 Dyscophus antongilii, AY691702 Ensatina eschscholtzi, AY583348 Euproctus asper, AY691709 Eurycea tonkawae, AY650122 Eurycea neotenes, AY691708 Eurycea quadridigitata, AY650121 Eurycea longicauda, AY691707 Eurycea multiplicata, AY691706 Eurycea bislineata, AY571649 Fejervaya sp., AY456255 Gegeneophis ramaswami, AY691710 Gyrinophilus porphyriticus, AY323764 Heleophryne regis, AY691712 Hemidactylium scutatum, AY887134 Hydromantes brunus, AY583340 Hymenochirus boettgeri, AY650144 Hynobius nebulosus,AY323769 Hyperolius viridiflavus, AY323772 Kaloula pulchra, AY887135 Karsenia koreana, AY650147 Ichthyophis sp, AY571652 Laliostoma labrosum, AY571653 Lankanectes corrugatu, AY583341 Lechriodus melanopyga, AY323771 Leptodactylus mystacinus, AY323770 Leptodactylus fuscus, AY323767 Litoria caerulea, AY723530 Mantella aurantiaca, AY723525 Mantella milotympanum, AY723528 Mantella pulchra, AY723520 Mantella madagascariensis, AY723519 Mantella betsileo, AY723529 Mantella viridis, AY723523 Mantella laevigata, AY723527 Mantella nigricans, AY723515 Mantella haraldmeieri, AY323774 Mantidactylus wittei, AY323760 Megophrys sp, AY323753 Mertensiella luschani, AY650136 Necturus alabamensis, AY323778 Nesomantis thomasseti, AY650134 Notophthalmus virides, AY691714 Nyctanolis pernix, AY571655 Nyctibatrachus major, AY650143 Onychodactylus japonicus, AY583351 Pachytriton labiatus, AY323758 Pelobates cultripes, AY583343 Pelodytes cf. punctatus, AY571656 Petropedetes parkeri, AY691700 Phaeognathus hubrichti, AY571657 Phrynomantis annectens, AY323761 Pipa parva, AY691718 Plethodon yonahlossee, AY691704 Plethodon ouachitae, AY691705 Plethodon serratus, AY691703 Plethodon cinereus,* ***L19324*** *Xenopus laevis, EF551562 Litoria ewingii, EF551561 AY323752 Ambystoma mexicanum,* ***AJ010258*** *Pleurodeles walt, AY691717 Plethodon welleri, EF551564 AY456257 Rhinatrema bivittatum, EF551566 Typhlonectes natans, EF551563 AY456256 Ichthyophis glutinosus, AY571661 Plethodontohyla alluaudi, AY571659 Polypedates dennysi, AY323777 Polypedates maculatus, AY323773 Pseudis paradoxa, AY650139 Pseudobranchus sp., AY650125 Pseudoeurycea rex, AY323776 Rana temporaria, AY650129 Rhyacotriton kezeri, AY650145 Salamandrella keyserlingii, AY323759 Scaphiopus couchii, AY456258 Scolecomorphus vittatus, AY691713 Stereochilus marginatus, AY650133 Taricha rivularis, AY583344 Telmatobius bolivianus, AY583354 Triturus marmoratus, AY456259 Uraeotyphlus cf. oxyurus.*

**Aves**

*AY228791 Actophilornis africanus, AY228792 Arenaria interpres, AY228796 Attagis gayi, AY228771 Burhinus vermiculatus, AY228777 Calidris canutus, AY228783 Catharacta skua, AF143736 Charadrius vociferus, AF143728 Chauna torquata, AF143737 Coracias caudata, AY625250 Euptilotis neoxenus, AY228775 Gallinago gallinago,* ***M58530*** *Gallus gallus, AF143732 Grus canadensis, AY625242 Harpactes erythrocephalus, AY443290 Hirundo rustica, AY056997 Hirundo pyrrhonota, AY228776 Jacana jacana, AY228773 Limosa haemastica, AF143731 Megapodius freycinet, AF143738 Passer montanus, AY228778 Phalaropus tricolor, AY625249 Pharomachrus pavoninus, AY228781 Phegornis mitchelli, AY228793 Pluvianellus socialis, AY625246 Priotelus rosiegaster, AY228767 Pterocles orientalis, AY228802 Scolopax rusticola, AY228803 Thinocorus orbignyanus, AY228785 Rissa tridactyla, AY228784 Rynchops niger, AY228786 Sterna eurygnatha, AY228772 Vanellus chilensis, AF143739 Tyrannus tyrannus, AY320006 Yuhina nigrimenta, AY319994 Paradoxornis gularis, AY056996 Garrulax milleti, AY319986 Macronous striaticeps, AY057027 Pycnonotus barbatus, AY056986 Cisticola anonymus, AY319998 Prinia bairdii, AY319992 Orthotomus sutorius, AY319972 Acrocephalus newtoni, AY319984 Hylia prasina, AY319977 Cettia brunnifrons, AY319997 Phylloscopus collybita, AY799821 Schoenicola brevirostris, AY319988 Megalurus palustris, AY319978 Cincloramphus mathewsi, AY320004 Thamnornis chloropetoides, AY319993 Panurus biarmicus, AY057035 Thraupis cyanocephala, AY057016 Parula americana, AY056998 Icterus parisorum, AY057022 Ploceus cucullatus, AY319983 Estrilda astrild, AY057007 Motacilla cinerea, AY057024 Prunella collaris, AY057028 Regulus calendula, AY443265 Catharus ustulatus, AY307215 Zoothera dauma, AY799820 Namibornis herero, AY057008 Muscicapa strophiata, AY307196 Luscinia cyane, AY056985 Cinclus cinclus, AY307186 Cinclus pallasii, AY307203 Oreoscoptes montanus, AY307197 Margarops fuscatus, AY319981 Dumetella carolinensis, AY307200 Mino anais, AY307193 Gracula religiosa, AY320000 Rhabdornis inornatus, AY319995 Phainopepla nitens, AY307204 Phainoptila melanoxantha, AY319980 Dulus dominicus, AY056981 Bombycilla garrulus, AY057030 Sitta pygmaea, AY443332 Sitta carolinensis, AY056983 Certhia familiaris, AY057038 Troglodytes aedon, AY057017 Parus inornatus, AY057009 Nectarinia olivacea, AY443261 Arcanator orostruthus, AY443301 Microeca papuana, AY443286 Eugerygone rubra, AY443339 Yuhina zantholeuca, AY056988 Coracina lineata, AY057012 Orthonyx spldingii, AY443273 Corcorax melanorhamphos, AY443296 Manucodia chalybata, AY443298 Melampitta lugubris, AY443297 Melampitta gigantea, AY443288 Grallina cyanoleuca, AY443280 Cyanocitta cristata, AY443293 Lanius excubitor, AY443289 Gymnorhina tibicen, AY443278 Cracticus quoyi, AY443270 Colluricincla harmonica, AY443308 Oriolus xanthonotus, AY443333 Sphecotheres viridis, AY443326 Ptilorrhoa caerulescens, AY443318 Pitohui cristatus, AY443287 Falcunculus frontatus, AY057010 Oedistoma iliolophum, AY057002 Melanocharis nigra, AY443299 Melanocharis versteri, AY443321 Pomatostomus halli, AY443269 Cnemophilus loriae, AY443317 Creadion carunculatus, AY057001 Malurus melanocephalus, AY057026 Ptilonorhynchus violaceus, AY443274 Cormobates leucophaeus, AY443294 Loboparadisaea sericea, AY443330 Schiffornis turdinus, AY443331 Scytalopus magellanicus, AY443264 Campylorhamphus trochilirostris, AY056995 Furnarius rufus, AY057021 Pitta guajara, AY443306 Neodrepanis coruscans, AY057018 Philepitta castanea*

**Chondrichthyes**

***U62645*** *Carcharinus leucas, AY949032 Ginglymostoma cirratum, AY462152 Carcharhinus plumbeus, AY462191 Triakis scyllium, AF135474 AY462145 Odontaspis ferox, AY949031 Negaprion brevirostris, AY949029 Rhinoptera bonasus, AY462188 Mustelus asterias, AY462185 Pseudotriakis microdon, AY462184 Proscyllium habereri, AY462179 Scyliorhinus canicula, AY462180 Scyliorhinus stellaris, AY462182 Scyliorhinus torazame, AY462178 Parmaturus sp., AY462174 Galeus murinus, AY462172 Cephalurus sp., AY462169 Asymbolus sp., AY462159 Apristurus longicephalus, AY462160 Apristurus manis, AY462161 Apristurus melanoasper, AF135483 Megachasma pelagios, AF135482 Carcharodon carcharias, AF135473 Alopias pelagicus, AF135481 Alopias superciliosus, AF135480 Isurus oxyrinchus, AF135479 Pseudocarcharias kamoharai, AF135478 Lamna ditropis, AF135477 Mitsukurina owstoni, AF135476 AY462147 Cetorhinus maximus, AF135475 Carcharias taurus*

**Crocodylia**

*AY239176 Tomistoma schlegelii, AF143724 AY125022 Alligator mississippiensis, AY239175 Osteolaemus tetraspis, AY239171 Alligator sinensis, AY239172 Crocodylus rhombifer, AY239173 Crocodylus intermedius, AY239174 Crocodylus cataphractus, AY239167 Caiman latirostris, AY239170 Paleosuchus trigonatus, AY239169 Paleosuchus palpebrosus, AY239166 Caiman crocodilus, AY239168 Melanosuchus niger, AF143725 Gavialis gangeticus*

**Lepidosauria**

*AY662639 Acontias meleagris, AY662619 Amphisbaena xera, AY662605 Anniella pulchra, AY662589 Anolis paternus, AY662620 Aspidoscelis tigris, AY662631 Asymblepharus sikimmensis, AY662599 Basiliscus plumifrons, AY444044 Bipes sp., AY662616 Bipes biporus, AY444050 Blanus strauchi, AY662577 Brookesia thieli, AY662584 Calotes calotes, AY662579 Calumma brevicornis, AY662604 Celestus enneagrammus, AY662638 Chalcides ocellatus, AY662578 Chamaeleo rudis, AY662643 Cordylus polyzonus, AY662627 Crenadactylus ocellatus, AY662630 Ctenotus robustus, AY662580 Ctenophorus salinarum, AY662613 Cylindrophis ruffus, AY662645 Dibamus sp., AY444053 Dibamus montanus, AY662611 Dinodon sp., AY662603 Elgaria panamintina, AY662593 Enyalioides laticeps, AY662615 Eremias sp., AY662622 Eublepharis turcmenicus, AY662634 Eumeces anthracinus, AY662633 Eumeces skiltonianus, AY662632 Eumeces inexpectatus, AY662629 Euprepis auratus, AY662637 Feylinia polylepis, AY662600 Gambelia wislizenii, AY662625 Gekko gecko, AY662614 Gloydius halys, AY662606 Heloderma suspectum, AY662592 Hoplocercus sp., AY662583 Hydrosaurus sp., AY662585 Japalura tricarinata, AY662609 Lanthanotus borneensis, AY662598 Leiocephalus cacarinatus, AY662587 Leiolepis belliana, AY662621 Leposoma parietale, AY662628 Lialis jicari, AY662595 Liolaemus pictus, AY444061 Loxocemus bicolor, AY662602 Ophisaurus attenuatus, AY662601 Oplurus cuvieri, AY662594 Phymaturus somuncurensis, AY662581 Physignathus lesueurii, AY662582 Physignathus cocincinus, AY662586 Phrynocephalus raddei, AY662590 Phrynosoma mcallii, AY662636 Proscelotes eggeli, AY662626 Pseudothecadactylus lindneri, AY524967 Rhampholeon kerstenii, AY524963 Rhampholeon brevicaudatus, AY524961 Rhampholeon nchisiensis, AY524955 Rhampholeon platyceps, AY524956 Rhampholeon chapmanorum, AY524953 Rhampholeon boulengeri, AY524952 Rhampholeon moyeri, AY524945 Rhampholeon sp, AY524943 Rhampholeon temporalis, AY524947 Rhampholeon marshalli, AY524949 Rhampholeon gorongosae, AY662612 Ramphotyphlops braminus, AY662618 Rhineura floridana, AY662591 Sauromalus ater, AY662635 Scelotes anguina, AY662610 Shinisaurus crocodilurus, AY662623 Sphaerodactylus shrevei, AY662597 Stenocercus crassicaudatus, AY662624 Teratoscincus przewalskii, AY662640 Typhlosaurus gariepensis, AY662641 Typhlosaurus lomii, AY662617 Trogonophis wiegmanni, AY662596 Uracentron flaviceps, AY662588 Uromastyx acanthinura, AY662608 Varanus griseus, AY662642 Xantusia vigilis, AY662607 Xenosaurus grandis, AY662644 Zonosaurus sp.*

**Mammalia**

***M29474*** *Homo sapiens,* ***M29475*** *Mus musculus,* ***AF305953*** *Lama glama,* ***AB091392*** *Sus scrofa,* ***M77666*** *Oryctolagus cuniculus,* ***U51897*** *Monodelphis domestica, DQ865896 Lutreolina crassicauda, AY294942 Apomys hylocoetes, DQ865883 Caluromys lanatus, DQ865884 Caluromys philander, DQ865887 Cryptonanaus unduaviens, DQ865886 Cryptonanaus chacoensis, AY294959 Neotoma floridana, DQ865888 Didelphis albiventris, DQ865890 Didelphis virginiana, EF551560 AY239195 AY125021 Elephas maximus, DQ865891 Glironia venusta, DQ865892 Gracilinanus aceramarcae, DQ865894 Gracilinanus emiliae, DQ865897 Marmosa lepida, DQ865900 Marmosa robinsoni, DQ865903 Marmosops incanus, DQ865904 Marmosops noctivagus, DQ865910 Micoureus regina, DQ865911 Monodelphis adusta, DQ865912 Monodelphis brevicauda, AY294951 Acomys ignitus, AY294946 Arvicanthis somalicus, EF551559 Ornithorhynchus anatinus, AY294943 Chrotomys gonzalesi, EF551555 AY125040 Notoryctes typhlops, AY294962 Irenomys tarsalis, AY294956 Cricetulus migratorius, AY294945 Mastomys natalensis, EF551558 AF303971 Tachyglossus aculeatus, EF551556 AY125037 Sarcophilus harrisii, AY834655 Artibeus jamaicensis, AF447520 Bos taurus, AB109345 Martes zibellina, AY065911 Alouatta seniculus, AF447507 Antrozous pallidus, AF203758 Cynopterus spinx, EF551557 AY125036 Cercartetus concinnus, AF447517 Desmodus rotundus, AY065913 Alouatta belzebul, AY065917 Ateles geoffroyi, AY065915 Ateles fusciceps, AY065919 Alouatta guariba, AY065910 Alouatta palliata, AY125028 AF447516 Equus caballus, AB109343 Martes foina, AB109344 Martes martes, AY834656 Anoura geoffroyi, AB109363 Leopardus pardalis, AF447521 Manis sp., AY834654 Macroderma gigas, AF203761 Felis catus, AB109355 Enhydra lutris, AB109340 Gulo gulo, AB109341 Martes americana, AF203760 Hipposideros commersoni, AB109342 Martes flavigula, AF203757 Megaderma lyra, AF447522 Megaptera novaeangliae, AB109356 Meles meles, AB109357 Melogale moschata, AB109362 Melursus ursinus, AB109358 Mephitis mephitis, AB109352 Mustela putorius, AB109348 Mustela eversmannii, AB109353 Mustela sibirica, AB109347 Mustela erminea, AB109350 Mustela nivalis, AB109354 Mustela vison, AF203754 Myotis daubentoni, AF447506 Nycteris grandis, AF447514 Nyctimene albiventer, AB109367 Paguma larvata, AB109365 Panthera pardus, AB109366 Panthera tigris, AB109364 Panthera leo, AB109360 Procyon cancrivorus, AB109359 Procyon lotor, AF203759 Pteropus rayneri, AF447508 Rhogeessa tumida, AF447515 Talpa europaea, AF203753 Tonatia bidens, AY834657 Thyroptera tricolor, AB109361 Ursus arctos.*

**Testudines**

*AY687907 Chelonia mydas, AY687908 Dermochelys coriacea, AY687924 Podocnemis expansa, AY687923 Pelusios williamsi, AY687922 Pelomedusa subrufa, AY687921 Chelodina longicollis, AY687904 Carettochelys insculpta, AY687906 Chelydra serpentina, AY687916 Graptemys pseudogeographica, AY687915 Trachemys scripta, AY687917 Actinemys marmorata, AY687914 Mauremys reevesii, AY687913 Heosemys spinosa, AY687912 Geochelone pardalis, AY687920 Elseya latisternum, AY687919 Phrynops gibbus, AY687918 Chelus fimbriatus, AY687911 Sternotherus odoratus, AY687910 Dermatemys mawii, AY687909 Staurotypus triporca, AY687905 Platysternon megacephalum, AY687903 Cyclanorbis senegale, AY687902 Lissemys punctata, AY687901 Apalone spinifera*
