# Supplementary material for: Base composition, selection, and phylogenetic significance of indels in the recombination activating gene-1 in vertebrates
Source: Front Zool. 2009 Dec 16;6:32. doi: 10.1186/1742-9994-6-32 (PMC2803162; doi:10.1186/1742-9994-6-32)
Supplement: Additional file 3 — Average nucleotide and amino acid distances. a) Average Tamura-Nei nucleotide distances, and average amino acid p-distances calculated for the 582 sequence dataset and for each part (5' and 3'-ends) of the entire fragment length of the gene. "N" indicates the number of sequences used. Check Table 1 to see the length range of the sequences included. "Bp" indicates the number of base pairs in the fragment analyzed after the within group alignment. Note that for Actinopterygii and Chondrichthyes only the 3' -end of the gene is provided. "F", "5"' and "3"' indicate the full gene and the 5' and 3' -ends of the gene, respectively.b) Average Tamura-Nei nucleotide distances, and amino acid p-distances for the functional zinc-finger and DNA binding domains in the 5' -end of the gene. "ZF" and "DB" indicate the zinc finger and DNA binding domains, respectively (Figure 1). See Methods for any additional information on the gene divisions and data available for each gene division. [file 1742-9994-6-32-S3.DOC]

**Additional file 3: Average nucleotide and amino acid distances**

a)

|  |  | **N** | **Bp** | **Mean** | **Mean 1^st^ cod. pos.** | **Mean 2^nd^ cod. pos** | **Mean 3^rd^ cod. pos** | **Mean aa distance** |
| --- | --- | --- | --- | --- | --- | --- | --- | --- |
| Actinopterygii | F | 110 | 3246 | 0.20 | 0.10 | 0.05 | 0.64 | 0.12 |
|  | 3’ | 110 | 1453-3246 | 0.20 | 0.10 | 0.05 | 0.64 | 0.12 |
| Amphibia | F | 113 | 3252 | 0.24 | 0.11 | 0.05 | 0.89 | 0.11 |
|  | 5’ | 7 | 1-1461 | 0.47 | 0.32 | 0.27 | 1.21 | 0.35 |
|  | 3’ | 113 | 1462-3252 | 0.24 | 0.11 | 0.05 | 0.89 | 0.11 |
| Aves | F | 119 | 3141 | 0.07 | 0.04 | 0.03 | 0.14 | 0.06 |
|  | 5’ | 119 | 1-1353 | 0.08 | 0.05 | 0.05 | 0.15 | 0.10 |
|  | 3’ | 119 | 1354-3141 | 0.05 | 0.03 | 0.01 | 0.13 | 0.02 |
| Chondrichthyes | F | 30 | 3336 | 0.11 | 0.07 | 0.03 | 0.28 | 0.08 |
|  | 3’ | 30 | 1360-3336 | 0.11 | 0.07 | 0.03 | 0.28 | 0.08 |
| Crocodylia | F | 13 | 2947 | 0.02 | 0.02 | 0.01 | 0.04 | 0.02 |
|  | 5’ | 13 | 1-1257 | 0.03 | 0.02 | 0.01 | 0.05 | 0.03 |
|  | 3’ | 13 | 1258-2947 | 0.02 | 0.01 | 0.00 | 0.04 | 0.01 |
| Lepidosauria | F | 84 | 2928 | 0.18 | 0.10 | 0.07 | 0.41 | 0.14 |
|  | 5’ | 80 | 1-1263 | 0.23 | 0.16 | 0.13 | 0.43 | 0.23 |
|  | 3’ | 84 | 1264-2928 | 0.15 | 0.07 | 0.03 | 0.40 | 0.08 |
| Mammalia | F | 89 | 3165 | 0.15 | 0.06 | 0.03 | 0.49 | 0.07 |
|  | 5’ | 37 | 1-1377 | 0.25 | 0.17 | 0.13 | 0.62 | 0.22 |
|  | 3’ | 89 | 1378-3165 | 0.15 | 0.05 | 0.03 | 0.48 | 0.06 |
| Testudines | F | 24 | 2793 | 0.06 | 0.04 | 0.02 | 0.13 | 0.04 |
|  | 5’ | 24 | 1-1212 | 0.07 | 0.05 | 0.03 | 0.14 | 0.07 |
|  | 3’ | 24 | 1213-2793 | 0.05 | 0.02 | 0.01 | 0.12 | 0.02 |

b)

|  |  | **N** | **Bp** | **Mean** | **Mean 1^st^ cod. pos.** | **Mean 2^nd^ cod. pos** | **Mean 3^rd^ cod. pos** | **Mean aa distance** |
| --- | --- | --- | --- | --- | --- | --- | --- | --- |
| Amphibia | ZF | 7 | 991-1110 | 0.28 | 0.17 | 0.08 | 0.78 | 0.19 |
|  | DB | 7 | 1294-1461 | 0.28 | 0.15 | 0.05 | 0.81 | 0.07 |
| Aves | ZF | 119 | 883-1002 | 0.05 | 0.02 | 0.02 | 0.11 | 0.04 |
|  | DB | 119 | 1186-1353 | 0.05 | 0.02 | 0.00 | 0.15 | 0 |
| Crocodylia | ZF | 13 | 787-906 | 0.02 | 0.01 | 0.00 | 0.04 | 0.01 |
|  | DB | 13 | 1090-1257 | 0.01 | 0.01 | 0.00 | 0.04 | 0.00 |
| Lepidosauria | ZF | 80 | 793-912 | 0.16 | 0.11 | 0.05 | 0.42 | 0.13 |
|  | DB | 80 | 1096-1263 | 0.14 | 0.08 | 0.01 | 0.47 | 0.03 |
| Mammalia | ZF | 37 | 913-1032 | 0.17 | 0.09 | 0.06 | 0.37 | 0.11 |
|  | DB | 37 | 1210-1377 | 0.17 | 0.09 | 0.02 | 0.53 | 0.06 |
| Testudines | ZF | 24 | 742-861 | 0.05 | 0.02 | 0.00 | 0.14 | 0.03 |
|  | DB | 24 | 1045-1212 | 0.06 | 0.04 | 0.00 | 0.15 | 0.00 |

**a)** Average Tamura-Nei nucleotide distances, and average amino acid p-distances calculated for the 582 sequence dataset and for each part (5’ and 3’ -ends) of the entire fragment length of the gene. “N” indicates the number of sequences used. Check Table 1 to see the length range of the sequences included. “Bp” indicates the number of base pairs in the fragment analyzed after the within group alignment. Note that for Actinopterygii and Chondrichthyes only the 3’ -end of the gene is provided. “F”, “5’” and “3’” indicate the full gene and the 5’ and 3’ -ends of the gene, respectively. **b)** Average Tamura-Nei nucleotide distances, and amino acid p-distances for the functional zinc-finger and DNA binding domains in the 5’ -end of the gene. “ZF” and “DB” indicate the zinc finger and DNA binding domains, respectively (Figure 1). See Methods for any additional information on the gene divisions and data available for each gene division.
