# Supplementary material for: Base composition, selection, and phylogenetic significance of indels in the recombination activating gene-1 in vertebrates
Source: Front Zool. 2009 Dec 16;6:32. doi: 10.1186/1742-9994-6-32 (PMC2803162; doi:10.1186/1742-9994-6-32)
Supplement: Additional file 4 — AT and GC skew. Average GC content (in % round up to higher integer) and base compositional skew for each codon position for each vertebrate group, based on the 582 sequences alignment. For the complete alignment across vertebrates 582 sequences have been used without the first 105 amino acid. See Methods for additional information about how the base compositional skew has been calculated. [file 1742-9994-6-32-S4.DOC]

**Additional file 4: AT and GC skew**

|  | **AT skew 1^st^ pos** | **AT skew 2^nd^ pos** | **AT skew 3^rd^ pos** | **GC content (%)** | **GC skew 1^st^ pos** | **GC skew 2^nd^ pos** | **GC skew 3^rd^ pos** |
| --- | --- | --- | --- | --- | --- | --- | --- |
| All vertebrates | +0.253 | +0.118 | -0.052 | 47 | +0.196 | -0.034 | +0.002 |
| Actinopterygii | +0.212 | +0.079 | -0.117 | 54 | +0.209 | -0.055 | -0.009 |
| Amphibia | +0.216 | +0.111 | -0.039 | 47 | +0.222 | -0.087 | -0.029 |
| Aves | +0.288 | +0.299 | -0.019 | 45 | +0.195 | -0.168 | +0.039 |
| Chondrichthyes | +0.216 | +0.116 | +0.026 | 43 | +0.285 | -0.042 | +0.157 |
| Crocodylia | +0.274 | +0.099 | -0.063 | 45 | +0.163 | -0.060 | +0.035 |
| Lepidosauria | +0.270 | +0.128 | -0.025 | 44 | +0.184 | -0.036 | -0.019 |
| Mammalia | +0.238 | +0.122 | -0.123 | 51 | +0.169 | -0.082 | -0.023 |
| Testudines | +0.287 | +0.128 | -0.027 | 46 | +0.190 | -0.066 | +0.013 |

Average GC content (in % round up to higher integer) and base compositional skew for each codon position for each vertebrate group, based on the 582 sequences alignment. For the complete alignment across vertebrates 582 sequences have been used without the first 105 amino acid. See Methods for additional information about how the base compositional skew has been calculated.
